# Severe Acute Respiratory Syndrome Coronavirus-2 Pandemia: Facts and Perspectives in a Bone Marrow Transplant Unit

**DOI:** 10.3389/fonc.2020.01626

**Published:** 2020-09-04

**Authors:** Michele Malagola, Nicola Polverelli, Lisa Gandolfi, Tatiana Zollner, Simona Bernardi, Camilla Zanaglio, Federica Re, Enrico Morello, Alessandro Turra, Alessandro Isidori, Domenico Russo

**Affiliations:** ^1^Bone Marrow Transplant Unit, Department of Clinical and Experimental Sciences, ASST Spedali Civili Brescia, University of Brescia, Brescia, Italy; ^2^CREA Laboratory (Hematological-Research AIL Centre), ASST-Spedali Civili Brescia, Brescia, Italy; ^3^Haematology and Haematopoietic Stem Cell Transplant Center, AORMN Hospital, Pesaro, Italy

**Keywords:** allogeneic stem cell transplanation, COVID 19, donor search algorithm, haploidentical donor, unrelated donor

Severe acute respiratory syndrome coronavirus-2 (SARS-CoV-2) pandemia and disease [coronavirus disease 2019 (COVID 19)] importantly changed the management of hematological patients (1), in particular those affected by acute leukemias, for whom allogeneic stem cell transplantation (allo-SCT) is a mainstay toward the cure of the disease (2).

Soon, after the progressive diffusion of the virus, most of the hematology units in Italy, following local hospitals and universities, started to modify the routine policies for diagnosis, hospitalization, and treatment of the underlying neoplasms. Any efforts for the rapid and correct diagnosis of the hematological disease continued to be made even during the pandemia, but undoubtedly, some changes in the decision algorithms have been considered to avoid infective risk for the patient. This is the case of deferrable treatments, such as those for indolent lymphomas or multiple myeloma in some cases, for which chemotherapy postponement can reduce the risk to be infected by SARS-CoV-2 and the incidence of treatment-related complications (e.g., neutropenia or immunosuppression), which can put the patient at risk to develop COVID 19. Other treatments, such as induction for acute leukemias or autologous stem cell transplantation for very high-risk lymphomas in complete remission (CR), are probably not easy to do be postponed without putting patients at high risk of dying for the underlying disease or of experiencing disease relapse. Allo-SCT is a life-saving treatment procedure for most of the patients, in particular for those who suffer from acute leukemias (2). As a consequence, deferring an allo-SCT should be considered very cautiously because a disease relapse in the meanwhile can make the patient no longer eligible for the transplant. Notably, the feasibility of allo-SCT strongly depends on donor availability too. This latter point is of interest because, during SARS-CoV-2 pandemia donors can get infected (and this excludes them from the donation) or can be worried to get infected (and thus withdrawal of the donation consent). Moreover, focusing on the matched unrelated donors (MUDs), stem cell transport from the donor centers to the transplant centers across the different countries, is expected to be extremely difficult during such a pandemia.

For these reasons, after the diffusion of SARS-CoV-2 across Italy, Europe, and the great majority of foreign countries, the Italian Authorities (Italian Ministry of Health and National Transplant Center) together with the Italian Group for Bone and Marrow Transplantation and the Italian Bone Marrow Donor Registry (IBMDR) rapidly faced the need to update, if not modify, the guidelines for donor selection and management. The same process was carried out in Europe by the European Group for Blood and Marrow Transplantation (3). As an example, as long as the transport of MUD stem cells across the countries seemed progressively more difficult, many Italian transplant centers adopted a policy of a preferential and fast selection of IBMDR donors or haploidentical donors. This was rapidly followed by the diffusion of institutional suggestions (promoted by IBMDR and Italian Group for Bone and Marrow Transplantation) to adopt this strategy as often as possible, also allowing cryopreservation of the donor stem cells, before conditioning starts.

What was the impact of this scenario on the daily clinical activity of our transplant center? First of all, we decided not to interrupt the transplant activity but to reduce it at the very beginning of the pandemia, to get familiar with the new virus and its consequences. Secondly, we modified the donor selection algorithm following the authorities' suggestions. Figure 1 shows the donor selection algorithm adopted in our transplant center before SARS-CoV-2 pandemia, to guarantee the transplant to the eligible patients within the ideal time according to the disease. This algorithm reflects the general observation that, currently, the time by which the transplant is performed is more important than the type of donor. This is particularly true in the setting of acute myeloid leukemias (AMLs), in which allo-SCT from sibling donors and well-matched unrelated donors have been demonstrated to be equally effective in the cure of the disease and equally safe, whereas transplants from partially matched unrelated donors have been demonstrated to perform significantly worse (4–6). In this context, the transplants from haploidentical donors with posttransplant cyclophosphamide have been compared with those from matched unrelated donors, and although some differences have been observed in the different studies, some in favor of haploidentical and others in favor of unrelated donors, no conclusive data are currently available. We recently retrospectively analyzed 110 patients allo-transplanted in our center from matched unrelated donors and 52 patients allo-transplanted from haploidentical donors between 2013 and 2019. The 1 year nonrelapse mortality, relapse incidence, and overall survival were 19 vs. 21% (*p* = 0.386), 33 vs. 16% (*p* = 0.342), and 60 vs. 64% (*p* = 0.513) for MUD vs. haploidentical allo-SCT, respectively (unpublished). Similarly, the cumulative incidences of acute grade II–IV graft vs. host disease and moderate–severe chronic graft vs. host disease at 1 year were 36 vs. 28% (*p* = 0.342) and 10 vs. 18% (*p* = 0.386), respectively (unpublished). Figure 2 shows how our donor selection algorithm changed after the onset of SARS-CoV-2 pandemia. Of note, we decided to maintain the MUD transplant program for a patient without an identical sibling, but we simultaneously decided to activate a transplant program from an alternative haploidentical donor, if available. Moreover, if a well-matched (10/10) unrelated donor had been identified, its workup would be activated only in case of a high probability of receiving the SCs successfully (e.g., IBMDR donor or MUD donor from a foreign country able to guarantee the exportation of SCs to Italy).

**Figure 1:**
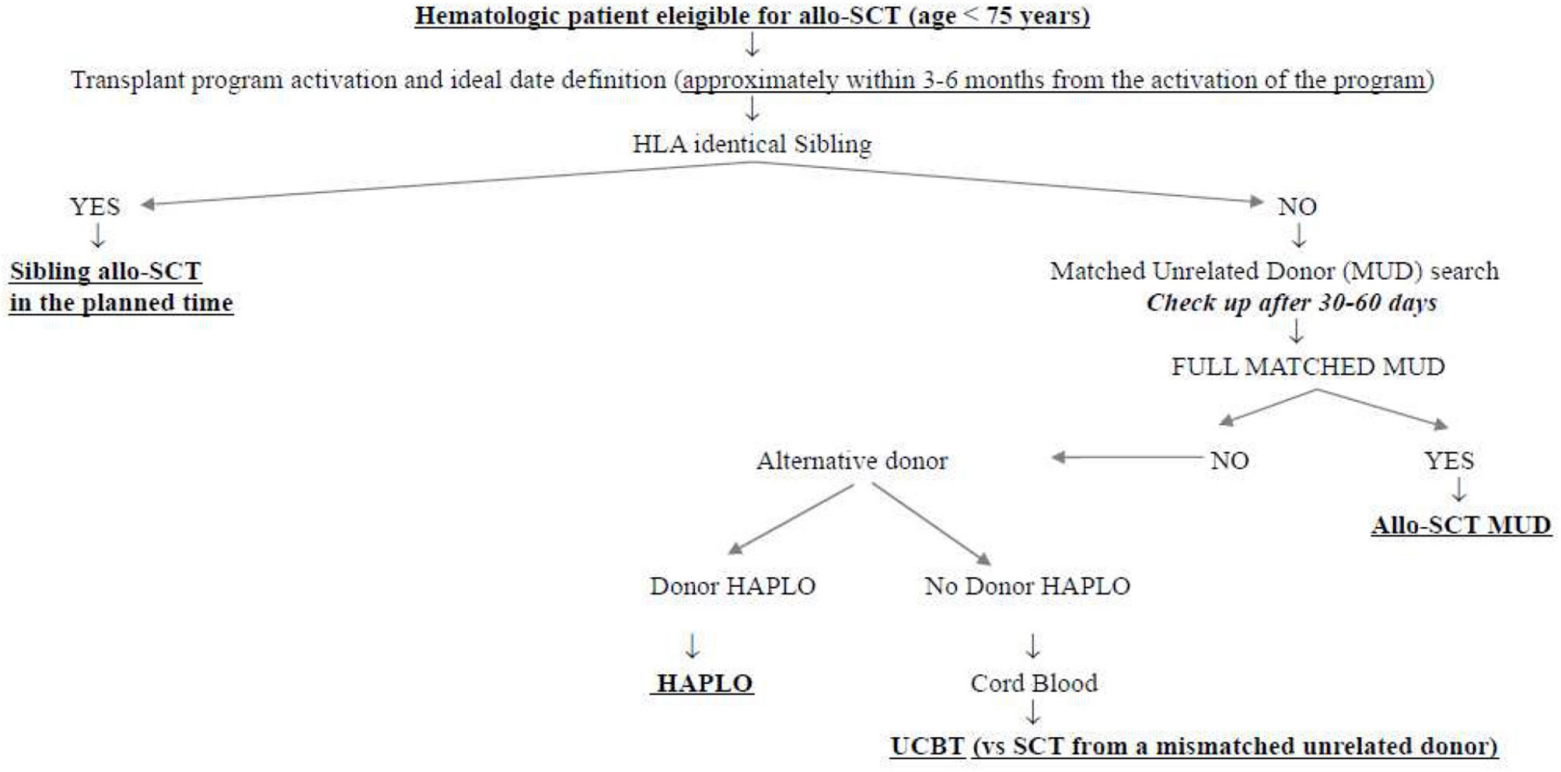
Algorithm for donor selection before SARS-CoV-2 pandemia.

**Figure 2:**
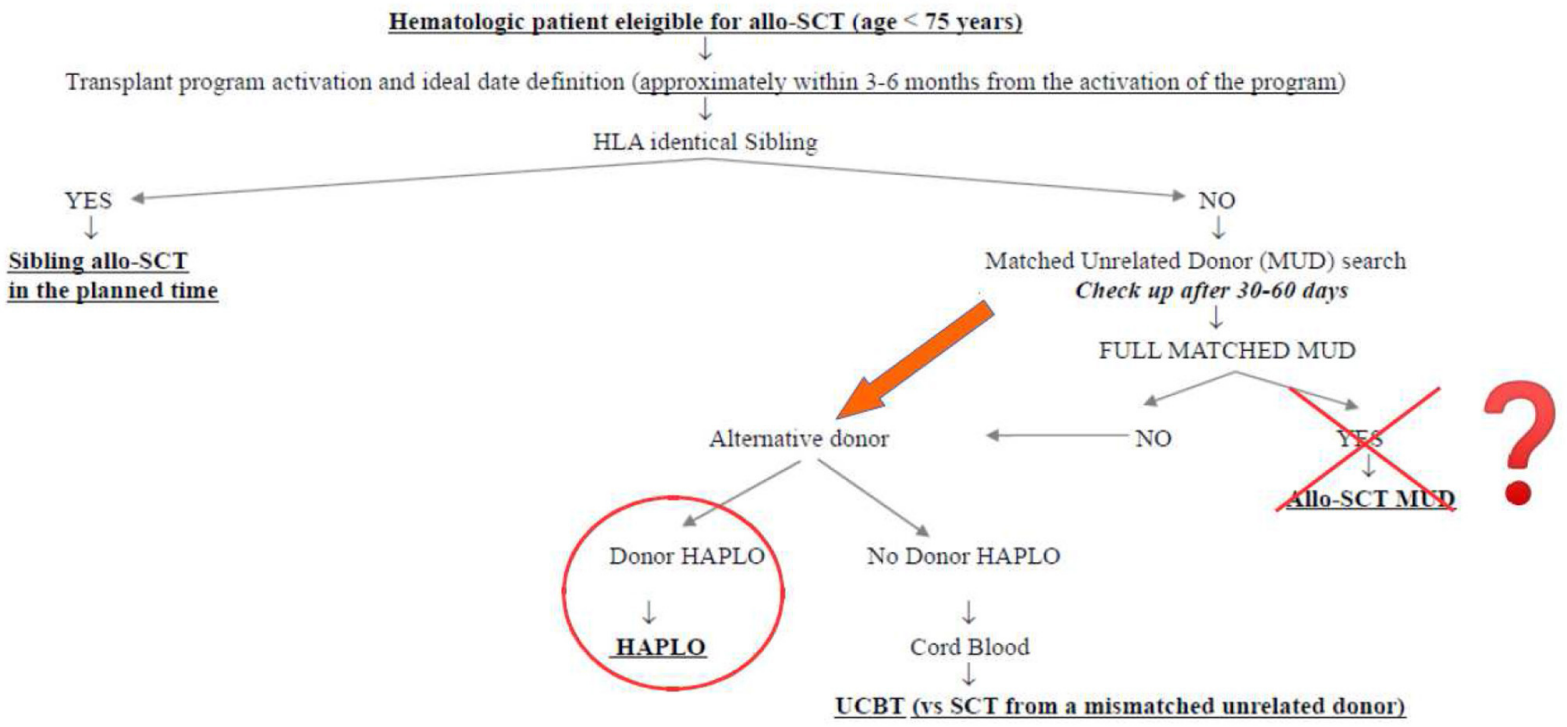
Algorithm for donor selection during SARS-CoV-2 pandemia.

In particular, between the 1st of February and the 15th of May, 2020, 14 allo-SCTs have been performed: seven AMLs, three multiple myelomas, two non-Hodgkin's lymphomas, one high-risk myelodysplastic syndrome, and one primary myelofibrosis. The median age of the patients was 50 years (range 35–66 years). All the patients with AML had a high-risk disease defined according to European LeukemiaNet published criteria (7) and were in CR at the time of allo-SCT. The three patients with multiple myeloma were in stringent CR according to the published criteria (8), whereas the two patients with non-Hodgkin's lymphoma had a chemosensitive relapse. The patients with high-risk myelodysplastic syndrome and primary myelofibrosis had stable disease at the time of allo-SCT. Three patients underwent transplantation from identical siblings, seven from MUDs, and four from haploidentical donors. Three of the four (75%) haploidentical transplants were performed in patients for whom the MUD search identified donors from countries from which the importation of stem cells was particularly impossible. All the transplants were performed using peripheral blood stem cells, and all the products of apheresis were cryopreserved before conditioning starts, after the donor's informed consent, to be confident of their collection. The median delay in each patients' transplant program concerning the planned timeline was 15 days (range 5–30 days). This delay was dependent on the need to have a negative SARS-CoV-2 test result before hospital admission and to discharge each patient very cautiously to minimize the risk of subsequent readmission for acute complications. The vitality on mononuclear cells checked 1 week after thawing on a separate vial was 98%.

We think that this scenario can teach us many important things. First, the transplant program of each patient has to be also maintained in such an emergency, providing coordinated teamwork, which involves the transplant centers, the donors' centers, and the collection centers, under the supervision of competent authorities (e.g., the IBMDR in Italy). Interestingly, 14 allo-SCTs have been performed in this critical timeframe, and this perfectly compares with the 14 allo-SCTs performed in the same period in 2019 (1st February−15th May 2019). This means that the efforts of the Italian transplant community were able to guarantee the prosecution of the transplant activity. Second, the activation of a fast and safe workup for haploidentical donors is highly preferable in comparison with a MUD, particularly if the unrelated donor comes from a foreign country. The safety and the outcome results of haploidentical transplants are almost comparable with those from MUD (3, 4), and we can speculate that the experience of SARS-CoV-2 pandemia could contribute to a progressive increase of the transplants from haploidentical donors and, conversely, to a reduction in the transplant from unrelated donors, worldwide. Third, even if we are now observing a reduction of infection rate, we think that the treatment algorithm reported in Figure 2 will be our clinical practice in the next months.

## Author Contributions

MM, NP, and DR wrote the opinion. All the authors gave their final approval before submission.

## Conflict of Interest

The authors declare that the research was conducted in the absence of any commercial or financial relationships that could be construed as a potential conflict of interest.
